# Supplementary material for: A Cell Double-Barcoding System for Quantitative Evaluation of Primary Tumors and Metastasis in Animals That Uncovers Clonal-Specific Anti-Cancer Drug Effects
Source: Cancers (Basel). 2022 Mar 8;14(6):1381. doi: 10.3390/cancers14061381 (PMC8946264; doi:10.3390/cancers14061381)
Supplement: Supplementary file 1 [file cancers-14-01381-s001.zip › cancers-1565005-supplementary.pdf]

Supplementary Information

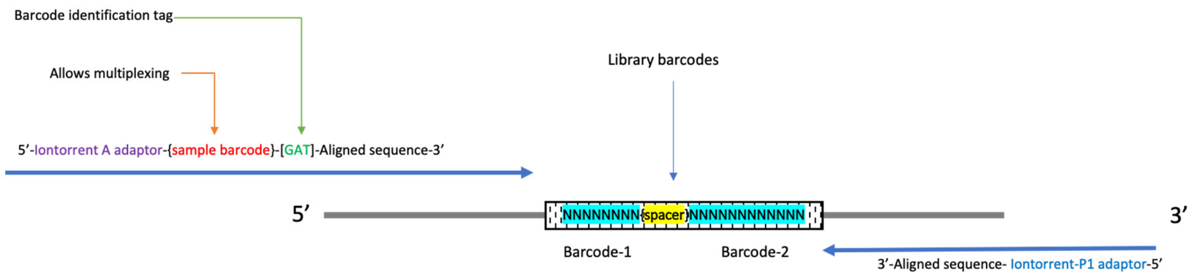

Figure S1. Map for primers for second round of nested PCR for 200K libraries common for different barcodes 1. Forward primer has sequence aligned to vector upstream of barcodes. The primer also contains an ion torrent adaptor A flanked at the 5' end, followed by a sample barcode for multiplexing samples that can be mixed together for sequencing purposes. Additionally, following sample barcodes, there is a sequence tag GAT that has been added to identify the place of sample barcodes in sequenced reads. The reverse primer contains aligned bases to vector map followed by ion torrent adaptor P1.

General information about DYRK1B inhibitor FX9847

The lead series of Felicitex DYRK1B inhibitors (FX) is based on a new anilino-thiazoloquinazoline scaffold. The structure of the lead compound FX9847 is shown in Figure S2.

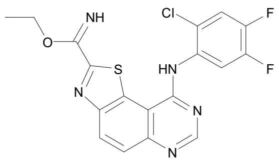

DYRK1B IC_{50} = 40 nM

Figure S2. FX9847 structure.

FX9847 is highly selective within the kinome. At 0.5 μM FX9847 inhibited to <10% of control 3 out of 403 wild type kinases tested (DiscoveRx KINOMEscan™): DYRK1B, DYRK1A, and Haspin. FX9847 is 100 times more potent against DYRK1B compared to DYRK1A or Haspin. Importantly, no kinases involved in cell cycle control, such as CDKs, were affected. FX9847 is permeable and is not a substrate of P-glycoprotein efflux pump and does not inhibit hERG. No toxicity following administration of FX9847 was observed in rodents at doses up to 12 mg/kg per day IP for 10 days.

Mechanism of action and proof of concept for FX9847 *in vitro*

Induction of pharmacological quiescence in NSCLC cells by an EGFR TKI and re-entry of H1975 cancer cells into the cell cycle following treatment with FX9847 is shown in Figure S3. We demonstrated that EGFR TK inhibitor osimertinib (Tagrisso®) an important new agent effective in more than half of erlotinib-resistant lung cancers, arrests cancer cells in G_0 phase (i.e. quiescence).

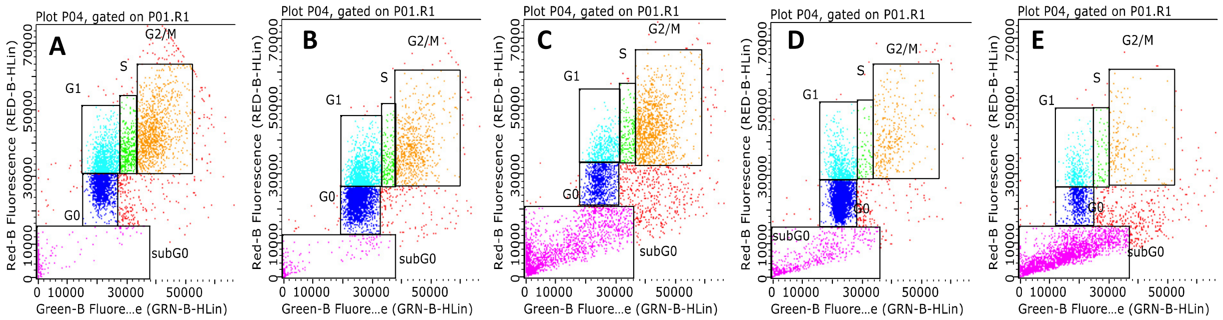

	Cell CycleDistribution, %				
	FBS+	FBS-	FX9847	Osimertinib	Osim+FX9847
**Sub-G_0**	3.1	2.4	35.2	11.9	62.1
**G_0**	29.0	47.5	17.2	62.5	12.6
**G_1**	25.4	27.5	9.5	13.6	8.2
**S**	7.6	4.2	4.8	1.5	1.7
**G_2/M**	25.2	14.0	23.2	6.6	4.7

Figure S3. Flow cytometry was used to quantitate the cell cycle distribution including G_0 phase of NSCLC H1975 cell. T790M. Cells were incubated for 48 hours with (A) Regular media; (B) FBS- media; (C) FX9847, 5 μM; (D) Osimertinib,18 nM; (E) Combination of FX9847, 5 μM and osimertinib, 18 nM. Corresponding population distributions within cell cycle are shown at the Table.

DYRK1B inhibitor, FX9847 forces cancer cells to re-enter cell cycle either when used alone or in combination with osimertinib. G_0 and G_1 phases of the cell cycle are differentiated based on their respective RNA content. Since G_0 cells are "quiescent", they exhibit lower gene transcription, thereby resulting in low RNA content compared to G_1 cells. Flow cytometry was used to quantify cell cycle distributions, following established techniques. NSCLC H1975 cells were labeled with acridine orange, which intercalates into RNA and into DNA, in which it fluoresces red and green, respectively. Treatment with osimertinib results in increase of cancer cells in G_0 phase: 62.5% (Figure S3D), compared to non-treated cells, 29% in G_0 (Figure S3A). There is also a 30% increase in quiescent cells after osimertinib treatment (Figure S3D) over quiescence induced by serum withdrawal (FBS-, Figure S3B). The combination of osimertinib and FX9847 results in cell cycle re-entry of NCLC H1975 cancer cells followed by induction of apoptosis: only 12% cells left

in G_0 (Figure S3E) after 24 hrs, and apoptosis (sub-G_0) was observed in 62% cells compared to 12% when treated with osimertinib alone. It is important to note that treatment with FX9847, either alone (Figure S3C) or in combination with osimertinib (Figure S3E) not only prevented the arrest of cells in G_0 as a consequence of osimertinib treatment, but also depleted pre-existent reservoir of quiescent cancer cells.

As expected, there was an accompanying increase in phosphorylation of DYRK1B substrate, Ser10-p27/Kip1 and an increase of the total p27/Kip1, since p27/Kip1 is stabilized by phosphorylation at Ser10 residue. An increase of the total p27/Kip1 levels is consistent with the observed G_0 arrest. The expression levels of both Ser10-p27/Kip1 and total p27/Kip1 decreased when H1975 cells were treated with a combination of FX9847 and osimertinib (lane 5) compared to osimertinib alone (lane 4). In addition, an increase in PARP cleavage confirms the induction of apoptosis when cells were treated with the combination of FX9847 and osimertinib versus osimertinib alone, consistent with the increase of sub-G_0 population observed by FACS analysis (Figure S3).

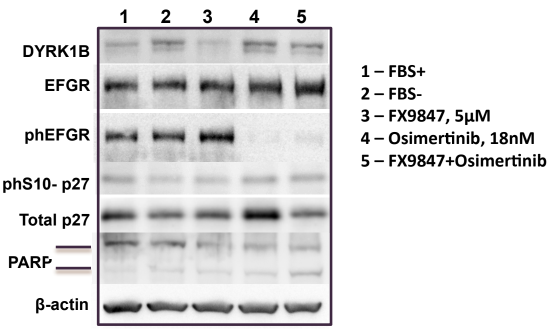

Figure S4. Western blot analysis of NSCLC H1975 cancer cells treated with vehicle control, FX9847, osimertinib, or the combination of these, as indicated, for 24 hours.

The proposed mechanism of action of the combination of DYRK1B inhibitor and EGFR TK inhibitor is the release of G_0 arrested cancer cells due to inhibition of the DYRK1B; decrease of the phosphorylation of its substrate, Ser10-p27 and the total p27/Kip1 levels that leads to cell cycle re-entry, and subsequently cancer cells' exposure to an EGFR TK inhibitor. That explains the observed significant increase in potency of EGFR TKIs (or decrease of the corresponding EC_{50} values for EGFR TKIs) when combined with DYRK inhibitor.

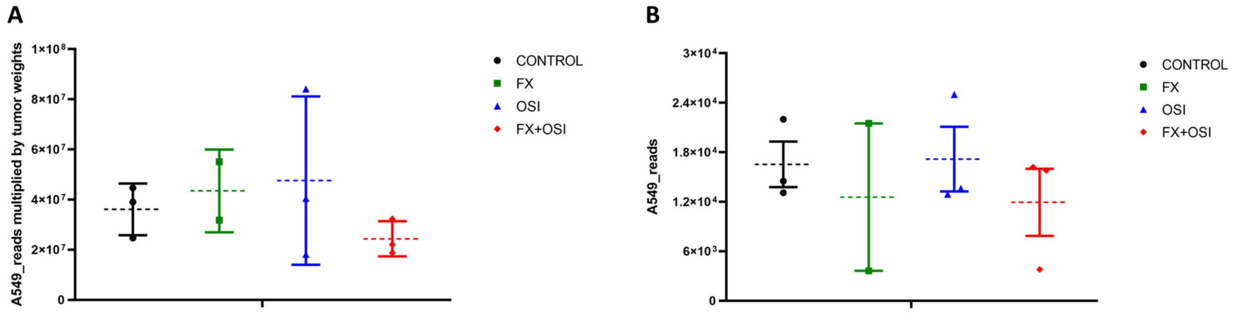

Figure S5. Effects of drug treatments on A549 in primary tumors and lung metastasis without normalization. A. A549 reads in primary tumor, normalized by tumor weights. B. A549 reads in lung metastasis.

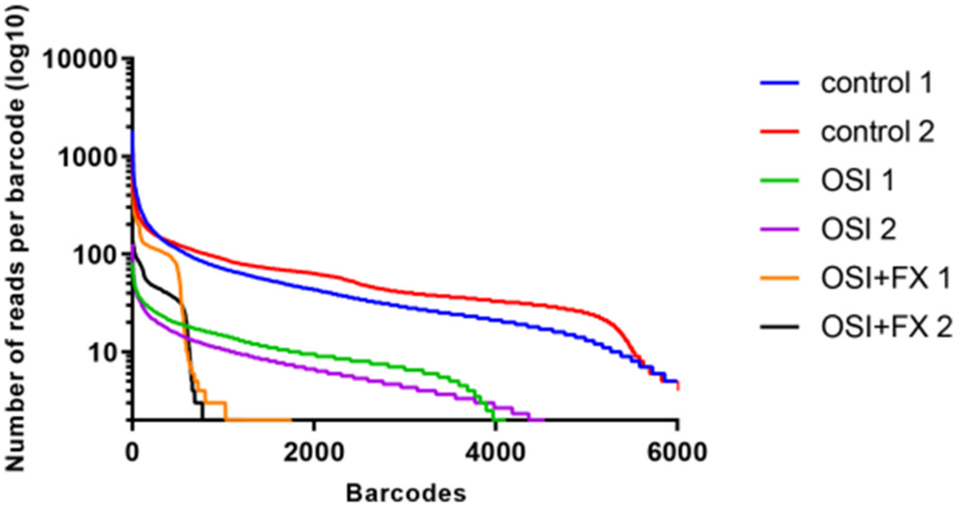

Figure S6. Reproducibility of distribution of barcodes in different animals. Distribution of barcodes in two control, two osimertinib-treated and two osimertinib+FX9847-treated animals is shown.

<u>Detailed procedure of PCR and primers</u>

Table S1. PCR primers of the 1-st round of nested PCR for amplification of barcodes for 200K libraries.

**Primer Name**	**Sequence 5` > 3`**
AVG_fw	CACGCTGTTTTGACCTCCATAGAA
AVG_rev_v2	GATCGCAGATCCTTCGCGGCC

Table S2. Primers for the second round of nested PCR for 200K library with different sample barcodes for the multiplexing purpose.

200K Barcode primers	Sequence 5` > 3`
AR_Bar1_IonForward	CCATCTCATCCCTGCGTGTCTCCGACTCAGTTGAGCCTATTCGATTTTTGACCTCCATAGAAGATTCTAGA
AR_Bar2_IonForward	CCATCTCATCCCTGCGTGTCTCCGACTCAGCATCATGGAACGATTTTTGACCTCCATAGAAGATTCTAGA
AR_Bar3_IonForward	CCATCTCATCCCTGCGTGTCTCCGACTCAGCTGGCAATCCTCGATTTTTGACCTCCATAGAAGATTCTAGA
AR_Bar4_IonForward	CCATCTCATCCCTGCGTGTCTCCGACTCAGCCGGAGAATCTAGATTTTTGACCTCCATAGAAGATTCTAGA
AR_Bar5_IonForward	CCATCTCATCCCTGCGTGTCTCCGACTCAGTCCACCTCCTAGATTTTTGACCTCCATAGAAGATTCTAGA
AR_Bar6_IonForward	CCATCTCATCCCTGCGTGTCTCCGACTCAGCAGCATTACCTCGATTTTTGACCTCCATAGAAGATTCTAGA
AR_Bar7_IonForward	CCATCTCATCCCTGCGTGTCTCCGACTCAGTCTGGCAACGATGATTTTTGACCTCCATAGAAGATTCTAGA
AR_Bar8_IonForward	CCATCTCATCCCTGCGTGTCTCCGACTCAGTCATAGAACACGATTTTTGACCTCCATAGAAGATTCTAGA
AR_Bar9_IonForward	CCATCTCATCCCTGCGTGTCTCCGACTCAGTCATTGATGTTCGATTTTTGACCTCCATAGAAGATTCTAGA
AR_Bar10_IonForward	CCATCTCATCCCTGCGTGTCTCCGACTCAGTCCTCCAAGTTCGATTTTTGACCTCCATAGAAGATTCTAGA
AR_Bar11_IonForward	CCATCTCATCCCTGCGTGTCTCCGACTCAGTGATAGATGTACGATTTTTGACCTCCATAGAAGATTCTAGA
AR_Bar12_IonForward	CCATCTCATCCCTGCGTGTCTCCGACTCAGGCGTTGATGATCGATTTTTGACCTCCATAGAAGATTCTAGA
AR_Bar13_IonForward	CCATCTCATCCCTGCGTGTCTCCGACTCAGAGCTTGTTCCTTGATTTTTGACCTCCATAGAAGATTCTAGA
AR_Bar14_IonForward	CCATCTCATCCCTGCGTGTCTCCGACTCAGGATATCAACTGGGATTTTTGACCTCCATAGAAGATTCTAGA
AR_Bar15_IonForward	CCATCTCATCCCTGCGTGTCTCCGACTCAGTCACTGCTCATCGATTTTTGACCTCCATAGAAGATTCTAGA
AR_Bar16_IonForward	CCATCTCATCCCTGCGTGTCTCCGACTCAGGAGCCGCTAAATGATTTTTGACCTCCATAGAAGATTCTAGA
AR_Bar17_IonForward	CCATCTCATCCCTGCGTGTCTCCGACTCAGATCTCCCTACTCGATTTTTGACCTCCATAGAAGATTCTAGA
AR_Bar18_IonForward	CCATCTCATCCCTGCGTGTCTCCGACTCAGCATCTGCTCTACGATTTTTGACCTCCATAGAAGATTCTAGA
AR_Bar19_IonForward	CCATCTCATCCCTGCGTGTCTCCGACTCAGGGCTCTGATAACGATTTTTGACCTCCATAGAAGATTCTAGA
AR_Bar20_IonForward	CCATCTCATCCCTGCGTGTCTCCGACTCAGTTCTGTCACGAAGATTTTTGACCTCCATAGAAGATTCTAGA
AR_Bar21_IonForward	CCATCTCATCCCTGCGTGTCTCCGACTCAGTGGTCCATCATCGATTTTTGACCTCCATAGAAGATTCTAGA

AR_Bar22_IonForward	CCATCTCATCCCTGCGTGTCTCCGACTCAGTCGTTCTCCCAAGATTTTTGACCTCCATAGAAGATTCTAGA
AR_Bar23_IonForward	CCATCTCATCCCTGCGTGTCTCCGACTCAGCAGACATTGACGGATTTTTGACCTCCATAGAAGATTCTAGA
AR_Bar24_IonForward	CCATCTCATCCCTGCGTGTCTCCGACTCAGATGGCGATCTAGGATTTTTGACCTCCATAGAAGATTCTAGA
AR_IonRev_P1	CCACTACGCCTCCGCTTTCCTCTCTATGGGCAGTCGGTGATGCGGCCGCGGATCCGATTTA

Table S3. Primers for the second round of nested PCR for 50M library with different sample barcodes for the multiplexing purpose.

50M Barcode primers	Sequence 5` > 3`
Sk_Bar1_IonForward	CCATCTCATCCCTGCGTGTCTCCGACTCAGGACTACGATCGGCATACGAAGACAGTTCG
Sk_Bar2_IonForward	CCATCTCATCCCTGCGTGTCTCCGACTCAGCAGACTGATCGGCATACGAAGACAGTTCG
Sk_Bar3_IonForward	CCATCTCATCCCTGCGTGTCTCCGACTCAGCATCGCGATCGGCATACGAAGACAGTTCG
Sk_Bar4_IonForward	CCATCTCATCCCTGCGTGTCTCCGACTCAGCACGCTGATCGGCATACGAAGACAGTTCG
Sk_IonRev_P1_v2	CCACTACGCCTCCGCTTTCCTCTCTATGGGCAGTCGGTGATTTGTATGTCTGTTGCTATTATGTCTAC

PCR reaction conditions

Table S4. Nested PCR reaction conditions for samples for 200K libraries.

**PCR 1**		**PCR 2**	
**Temperature**	**Cycles**	**Temperature**	**Cycles**
95°C, 2 minutes	1 cycle	98°C, 30 seconds	1 cycle
95°C, 30 seconds	28-40 cycles [1] or 31-40 cycles [2]	98°C, 10 seconds	5-12 cycles [1] or 7-8 cycles [2]
68°C, 20 seconds		65°C, 15 seconds	
68°C, 15 seconds		72°C, 15 seconds	
68°C, 1.5 minutes	1 cycle	72°C, 5 minutes	1 cycle

[1] for Tumor samples

[2] for Lung and Liver samples

50M library amplification

Table S5. Nested PCR reaction conditions for samples from cell line for 50M libraries.

**PCR-1**		**PCR-2**	
**Temperature**	**Cycles**	**Temperature**	**Cycles**
94°C, 2 minutes	1 cycle	94°C, 2 minutes	1 cycle
94°C, 45 seconds	21 cycles	94°C, 45 seconds,	8 cycles
65°C, 15 seconds		65°C, 15 seconds,	
72°C, 30 seconds		72°C, 30 seconds	
72°C, 2 minutes	1 cycle	72°C, 2 minutes	1 cycle
